# Transgenic *Plasmodium berghei* expressing *P. vivax* pre-erythrocytic antigens SSP3 and SPECT1 to elucidate their efficacy towards vaccine development

**DOI:** 10.1371/journal.pntd.0014810

**Published:** 2026-10-22

**Authors:** Surendra Kumar Kolli, Pradeep Annamalai Subramani, Jai Ramesar, Justin Nicholas, Madison M. Ogbondah, Samantha J. Barnes, Pooya Mahdavi, Ahmed M. Salman, Francis Babila Ntumngia, Chris J. Janse, John H. Adams

**Affiliations:** 1 Center for Global Health and Interdisciplinary Research, College of Public Health, University of South Florida, Tampa, Florida, United States of America; 2 Leiden University Center for Infectious Diseases (LUCID), Leiden University Medical Center, Leiden, Netherlands; 3 Nuffield Department of Medicine, The Jenner Institute, University of Oxford, Oxford, United Kingdom; National University of Singapore, SINGAPORE

## Abstract

The development of an efficacious vaccine against *Plasmodium vivax* malaria remains a top global health priority. *P. vivax* forms hypnozoites, dormant forms of the parasite in the liver that can become reactivated after a few months or even years, causing relapse malaria and enhancing transmission. Therefore, targeting antigens expressed during the pre-erythrocytic stages offers the potential to prevent clinical malaria from both primary and relapse infections. The circumsporozoite protein (CSP) is the leading pre-erythrocytic vaccine candidate and forms the basis for the current *P. falciparum* malaria vaccines RTS, S and R21/Matrix M. Studies have shown that in *P. vivax*, CSP attributes limited protection, highlighting the need to identify additional pre-erythrocytic antigens to improve vaccine efficacy. Evaluation of new candidates often requires human volunteers, making initial screening complex and highlighting the need for robust preclinical models. In this study, we generated transgenic *P. berghei* parasite lines as research tools to investigate the potential efficacy of two *P. vivax* pre-erythrocytic vaccine candidates, SSP3 and SPECT1. The chimeric *Pb-Pv*SSP3 and *Pb-Pv*SPECT1 lines showed normal development throughout the parasite life cycle, in both mice and mosquitoes. Antisera raised against recombinant *Pv*SSP3 and *Pv*SPECT1 using TitreMax Gold adjuvant exhibited reduction in sporozoite invasion of hepatocytes *in vitro*, with mean inhibitions of 27% and 14%, respectively. *In vivo* sporozoite challenge studies showed sterile protection in 10% of mice immunized with r*Pv*SPECT1, whereas r*Pv*SSP3 provided no detectable protection. Although substantial protection was not observed for either antigen under the conditions tested, the transgenic *P. berghei* lines generated in this study serve as useful platforms for evaluating the efficacy of these antigens as vaccine candidates across different delivery systems and adjuvanted formulations.

## Author summary

*Plasmodium vivax* is the most geographically widespread human malaria parasite outside Africa. It presents a unique challenge by forming dormant liver stages called hypnozoites that can reactivate, leading to relapse of blood-stage infections and mosquito transmission. This complicates elimination efforts and maintains transmission reservoirs. Focusing on pre-erythrocytic vaccines that target the sporozoite and liver stages of *P. vivax* offers a promising strategy to prevent establishing the initial liver stage infection and reduce disease transmission. However, progress in developing a pre-erythrocytic *P. vivax* vaccine has been slow because of the lack of easily attainable laboratory-produced sporozoites to test its efficacy. Fortunately, transgenic rodent malaria parasites expressing *P. vivax* antigens provide reproducible challenge models for assessing vaccine-induced immunity in mice. In this study, we evaluated the efficacy of two novel *P. vivax* pre-erythrocytic candidates, SSP3 and SPECT1, using transgenic *P. berghei* strains expressing *P. vivax* SSP3 and SPECT1 by gene replacement. *Pv*SSP3 and *Pv*SPECT1 antisera inhibited hepatocyte invasion by transgenic sporozoites *in vitro*. However, preliminary results from *in vivo* challenge experiments provided 10% sterile protection with *Pv*SPECT1 but no significant protection for *Pv*SSP3. The transgenic *P. berghei* lines generated in this study can be used to evaluate the efficacy of these antigens through different delivery systems and adjuvanted vaccine formulations.

## Introduction

Malaria is caused by an apicomplexan species, *Plasmodium,* and is responsible for 282 million cases and 610,000 deaths in 2024 alone [1]. *P. vivax* causes less severe malaria than *P. falciparum*, but is sometimes fatal and it is widespread outside sub-Saharan Africa, particularly in Southeast Asia, Latin America, and parts of the Western Pacific [2]. Activation of *P. vivax* forms dormant liver-stage parasites, called hypnozoites, can lead to a new blood-stage infection, termed relapse, months or years after the infecting mosquito bite [3]. In *P. vivax*, the transmission stages called gametocytes appear immediately with initial blood-stage infection, facilitating transmission before clinical symptoms develop and hindering efforts to control vivax malaria at the population-level [4,5]. In addition, the potential spread of drug-resistant *P. vivax* strains poses a major barrier to malaria elimination and highlights the importance of vaccine development [6].

The development of pre-erythrocytic (PE) malaria vaccines offers the potential for achieving sterile protective immunity [6,7]. The clinically silent PE stages encompass the journey of motile sporozoites lodged in the dermis by a female anopheline mosquito, until the first-generation merozoites are released into the blood [8–10]. Targeting this PE phase of the life cycle is critical because it can prevent clinical disease. In the case of *P. vivax*, developing an effective PE vaccine is crucial as it prevents the formation of dormant hypnozoites and subsequent relapses [6,11,12]. Currently, there are two licensed malaria vaccines, RTS,S (Mosquirix) and R21/Matrix-M. Both target the *P. falciparum* circumsporozoite protein (CSP) and elicit anti-CSP antibody responses associated with protection [13,14]. RTS,S has demonstrated moderate efficacy against clinical malaria in children, although protection wanes over time [13–16]. The R21/Matrix-M has shown higher efficacy in clinical trials, with around 75–80% protection against clinical malaria in children [14,17]. However, these *P. falciparum*-licensed vaccines do not provide cross-protection against *P. vivax* or other *Plasmodium* species [18]. The CSP in *P. vivax* has three allelic variants (VK210, VK247, and *P. vivax*-like) that differ in the B-cell immunodominant central repeat region [19]. A recombinant chimeric repeat-version of *P. vivax* CSP is the only *P. vivax* PE vaccine that has been clinically tested and found to be safe, but failed to confer protection in human trials [20]. The other *P. vivax* pre-erythrocytic vaccine candidates explored so far in preclinical studies are TRAP and CelTOS. *P. vivax* TRAP showed partial protection in *Aotus* and measurable efficacy in mice [21,22]. In contrast, *P. vivax* CelTOS showed weaker, less consistent efficacy. *Pv*CelTOS is immunogenic during natural infections with responses persisting up to 1 year, and the antisera inhibited sporozoite invasion of human hepatocytes [7,23,24]. Despite being immunogenic across several vaccination platforms, *Pv*CelTOS provided modest protection in viral vectored vaccine models [25]. Therefore, there is a need to identify complementary PE antigens for inclusion in a more robust, multistage, multivalent vaccine formulation that could provide broad, efficacious, potentially long-lasting protection and reduce transmission [6,12].

The basis for selecting novel PE antigens for an antibody-based vaccine must be to identify targets on the sporozoite surface that are essential for infectivity, immunogenic, and unique to *Plasmodium* [26]. When an infected mosquito deposits *Plasmodium* sporozoites into the dermis, they glide rapidly, locate nearby blood vessels, and enter the circulation to reach the liver [27]. In the liver, sporozoites cross the sinusoidal barrier, traverse multiple cells, and then productively invade a hepatocyte, forming a parasitophorous vacuole (PV) to establish infection [9,28]. Multiple sporozoite proteins are known to have critical functions in PE phases of gliding motility, cell traversal, and liver infection [8,29] and these functionally critical proteins are considered more vulnerable PE vaccine targets to block infection.

Systematic proteomic and transcriptional profiling have identified several sporozoite surface proteins that are essential for establishing liver-stage infection as promising PE vaccine candidates [30–33]. We selected sporozoite surface protein 3 (SSP3) and sporozoite microneme protein essential for cell traversal (SPECT1), which are upregulated upon encountering a mammalian-like environment [32]. Importantly, both *P. vivax* PE antigens have also demonstrated high seroprevalence after natural infections and can elicit functionally inhibitory antibodies that can block sporozoite infection and development of liver stages [7,23].

SSP3 is a type I transmembrane protein localized on the sporozoite surface that plays a vital role in gliding motility and in the maturation of exoerythrocytic forms (EEFs) [34,35]. Interestingly, SSP3 appears to be unmasked once the immunodominant CSP is shed during invasion, making it an accessible target for inhibitory antibodies [34]. SPECT1 is a microneme-derived protein essential for the parasite’s ability to migrate through host cells, such as Küpffer cells and the liver sinusoidal wall [28]. Structural studies indicate that SPECT1 has 4 - alpha helices (α1 to α4) and a hook structure made from two antiparallel short helices α3a and α3b [36]. Its involvement in host cell traversal extends beyond hepatocytes to macrophages, suggesting a broader role in establishing infection [37].

Establishing a robust, continuous *in vitro* blood-stage culture system for *P. vivax* remains elusive. As a result, access to *P. vivax* sporozoites remains limited to human or primate infections, creating a bottleneck to evaluating the efficacy of the pre-erythrocytic vaccine candidates. To overcome the constraints of the limited availability of *P. vivax* sporozoites, the transgenic *P. berghei* (rodent malaria) model serves as a valuable substitute for evaluating the efficacy of pre-erythrocytic vaccine candidates *in vitro* and *in vivo* [38–40]. These transgenic parasites are created by replacing the endogenous *P. berghei* genes with their *P. vivax* orthologs. This strategy enables the preclinical assessment of the protective efficacy of human malaria antigens in reproducible *in vivo* and *in vitro* platforms. This model enables the comparative assessment of antigen performance within different delivery platforms and facilitates testing various adjuvants to identify formulations that elicit stronger, more durable immune responses.

In this study, we generated chimeric *P. berghei* parasites in which the endogenous *ssp3* and *spect1* genes were replaced with the *P. vivax* orthologs. To validate that these gene replacements would generate viable parasites capable of propagating in mice and mosquitoes, we assessed asexual blood-stage propagation and transmissibility to mosquitoes by quantifying oocysts and sporozoites. The infectivity of sporozoites was assessed by *in vitro* liver-stage development, quantification of the *in vivo* liver burden, and the pre-patency period. We assessed the functional activity of antigen-specific antisera generated in our previous study [7] by an *in vitro* inhibition of liver-stage development assay (ILSDA) and, as a proof-of-concept study, evaluated vaccine-induced protection following sporozoite challenge in mice by intravenous (i.v.) injection. Together, these studies establish *Pb-Pv*SSP3 and *Pb-Pv*SPECT1 transgenic parasites for preclinical evaluation of different vaccine-delivery platforms and adjuvant formulations.

## Methods

### Ethical statement

At Leiden University Medical Center, all animal experiments were granted with a license by Competent Authority after ethical evaluation by the Animal Experiments Committee Leiden (AVD1160020171625). All experiments were performed in accordance with the Experiments on Animals Act (Wod, 2014), the applicable legislation in the Netherlands, in accordance with the European guidelines (EU directive no. 2010/63/EU) regarding the protection of animals used for scientific purposes. At the University of South Florida, all animal studies were performed in adherence to the regulations of the Institutional Animal Care and Use Committee (IACUC), IACUC # IS00008179.

### Experimental animals and *P. berghei* reference lines

Six- to eight-week-old female Swiss Webster ND4 (outbred) mice, female BALB/c (inbred) mice (Envigo, USA) and female OF1 (outbred) mice (6–7 weeks; Charles River, NL) were used in the current study. All experiments were executed in a licensed establishment for the use of experimental animals. Mice were housed in individually ventilated cages furnished with autoclaved aspen woodchip, fun tunnel, wood chew block, and nestlets at 21 ± 2°C under a 12:12 h light-dark cycle at a relative humidity of 55 ± 10%.

*P*. *berghei* ANKA mCherry reporter parasite line 1868cl1 (RMgm-1320, http://www.pberghei.eu) was used to generate *Pb-Pv*SSP3 transgenic line, and *P*. *berghei* ANKA GFP reporter parasite line 676m1cl1 (RMgm-29; www.pberghei.eu) was used to generate *Pb-Pv*SPECT1 transgenic line. The 1868cl1 reporter line expresses *mCherry* and *luciferase* reporters under the constitutive *Pbhsp70* and *Pbeef1α* promoters, respectively, that were integrated into the neutral *230p* gene locus [41]. The 676m1cl1 reporter line expresses a *gfp-luciferase* fused gene under the constitutive *Pbeef1α* promoter, which was integrated into the neutral *230p* gene locus [42].

### Generation of transgenic *P. berghei* parasites

The transgenic *P. berghei* parasite lines expressing *Pv*SSP3 (PVP01_1427900) or *Pv*SPECT1 (PVP01_1212300) were generated following a 2-step GIMO transfection protocol [43]. To generate the transgenic line expressing *Pv*SSP3, the *Pbssp3 open reading frame* (*orf*) was first replaced with a positive-negative drug selectable marker (SM), h*dhfr::*y*fcu,* to generate the *Pb*SSP3-GIMO line. This was achieved by creating a DNA targeting construct, pL2381. The *Pbssp3 5’* and *3’* homology regions were amplified using the primers JA-4374/JA-4375 and JA-4376/JA-4377 (S1 Table shows the primer sequences), respectively, and introduced into pL0034 using the restriction sites HindIII/SacII and KpnI/NotI, respectively, to obtain pL2381. The plasmid pL2381 was linearized with restriction enzymes HindIII/NotI and transfected into the 1868cl1 parasite line following standard protocol [44]. The *Pb*SSP3-GIMO parasites (line 3374) were selected by providing pyrimethamine in the drinking water of mice *ad libitum* [44] and cloned by limiting dilution [45]. The correct integration of the targeting constructs was validated by diagnostic PCR analysis of gDNA and Southern blot hybridization analysis of PFG-separated chromosomes as described previously [44]. The primers used for PCR genotyping are listed in S1 Table. Next, the h*dhfr*::*yfcu* positive-negative selectable marker cassette in *Pb*SSP3-GIMO was replaced with *Pvssp3 orf* by GIMO transfection to create a transgenic *Pb-Pvssp3* replacement line. This was achieved by generating a DNA targeting construct, pL2390. The *Pvssp3 orf* was amplified from *P. vivax* genomic DNA isolated from cryopreserved *P. vivax* VK247-positive sporozoites [4] using the primers JA-4439/JA-4440 (S1 Table). The primer JA-4439 has 27 bp of *Pbssp3 5’ utr* upstream to the start codon, and the primer JA-4440 has 21 bp of *Pbssp3 3’ utr* downstream to the stop codon*.* The plasmid pL2381 was digested with SacII and KpnI restriction sites, and *Pvssp3 orf* was introduced in place of the h*dfhr*::y*fcu* cassette using NEBuilder (New England Biolabs) following the manufacturer’s instructions to obtain pL2390. The plasmid pL2390 was linearized with restriction enzymes HindIII/NotI and transfected into 3374cl1 parasites following standard protocol [44]. The *Pb-Pv*SSP3 parasites (line 3395) were selected by providing 5-FC in the drinking water of mice [44].

To generate the transgenic line expressing *Pv*SPECT1, the *Pbspect1 orf* was first replaced with a positive-negative SM, h*dhfr::*y*fcu,* to generate the *Pb*SPECT1-GIMO line. This was achieved by creating a DNA targeting construct, pL2157. The *Pbspect1 5’* and *3’* homology regions were amplified using the primers AS-1256/AS-1257 and AS-1258/AS-1259 (S1 Table shows the primer sequences), respectively, and introduced into pL0034 using the restriction sites ApaI/SacII and XmaI/NotI, respectively, to obtain pL2157. The plasmid pL2157 was linearized with restriction enzymes ApaI/NotI and transfected into the 676m1cl1 parasite line following standard protocol [44]. The *Pb*SPECT1-GIMO parasites (line 2672) were selected by providing pyrimethamine in the drinking water of mice [44] and cloned by limiting dilution [45]. The correct integration of the targeting constructs was validated by diagnostic PCR analysis of gDNA and Southern blot hybridization analysis of PFG-separated chromosomes as described previously [44]. The primers used for PCR genotyping are listed in S1 Table. Next, the h*dhfr*::*yfcu* positive-negative selectable marker cassette in *Pb*SPECT1-GIMO was replaced with *Pvspect1 orf* to generate a transgenic *Pb-Pvspect1* replacement line. This was achieved by generating a DNA targeting construct pL2383. The *Pvspect1 orf* was amplified from *P. vivax* genomic DNA using the primers JA-4612/JA-4613 (S1 Table). The *Pvspect1 orf* fragment was purified and introduced into the SacII/XhoI restriction sites of pL2157 plasmid, replacing the h*dhfr*::*yfcu* cassette to obtain pL2383. The plasmid pL2383 was linearized with restriction enzymes ApaI/NotI and transfected into 2672cl2 parasites following standard protocol [44]. The *Pb-Pv*SPECT1 parasites (line 3378) were selected by providing 5-FC in the drinking water of mice [44]. *Pb-Pv*SSP3 (3395) and *Pb-Pv*SPECT1 (3378) parasites were cloned by limiting dilution [45], and a clone of each parasite line was selected for the current study. The correct integration of the targeting constructs was analyzed by diagnostic PCR of gDNA and Southern blot hybridization of PFG-separated chromosomes as previously described [44]. The primers used for PCR genotyping are listed in S1 Table.

### Phenotype analysis of the transgenic lines

The asexual multiplication rate of *Pb-Pv*SSP3 and *Pb-Pv*SPECT1 parasites in mice was determined during the cloning procedure [46]. To analyze the transmission and development of *Pb-Pv*SSP3 and *Pb-Pv*SPECT1 transgenic parasites in the mosquitoes, 3–5 day old female *Anopheles stephensi* were fed on anesthetized female Swiss Webster ND4 mice that harbor at least one gametocyte per field in a Giemsa-stained thin blood smear. The infected mosquitoes were maintained at 21.5 °C and 80% relative humidity and provided 10% glucose with 0.05% 4-aminobenzoic acid (Sigma) ad libitum. To monitor successful parasite transmission, infected mosquito midguts were examined for oocysts on day 14 post feeding. The salivary glands of infected mosquitoes were dissected on days 18–21 post feeding to isolate salivary gland sporozoites (Sg-Spz). The dissected salivary glands were collected in 100 µl of Leibovitz’s medium (Thermo Fisher Scientific) in a 1.5 ml microfuge tube and centrifuged at 3000 rpm for 1 min. The glands were mechanically disrupted by crushing with a plastic pestle to release the sporozoites. The sporozoite suspension was filtered using a 40 µm cell strainer (Greiner Bio-One, USA), and sporozoites were counted using a hemocytometer. To determine the infectivity of Sg-spz, 1x10^4^ salivary gland sporozoites were injected into mice by intravenous injection, and the parasite liver burden was quantified at 44 h post infection using IVIS Lumina II Imaging System (Perkin Elmer Life Sciences, Waltham, USA). Starting on day 4 post-infection, parasitemia in the mice was measured by counting parasites against at least 10,000 RBCs in Giemsa-stained tail blood smears. The prepatent period is defined as the time to reach 1% parasitemia.

#### Recombinant protein production.

Recombinant protein expression and purification of *Pv*SSP3 and *Pv*SPECT1 were performed as previously reported [7]. In brief, amino acids 19–203 of *Pv*SSP3 and full-length SPECT1 (without signal peptide), eliminating the stop codon (Fig 1A and 1B), were cloned into the pET21a+ expression vector that has a C-terminal hex-histidine tag. Recombinant *Pv*SSP3 and *Pv*SPECT1 were expressed in OneShot BL21 Star (DE3) *E. coli* cells (Thermo Fisher Scientific) and purified using His-Trap HP affinity chromatography using the Akta pure system (Cytiva) and the purity of r*Pv*SSP3 and r*Pv*SPECT1 was assessed by SDS-PAGE.

**Fig 1:**
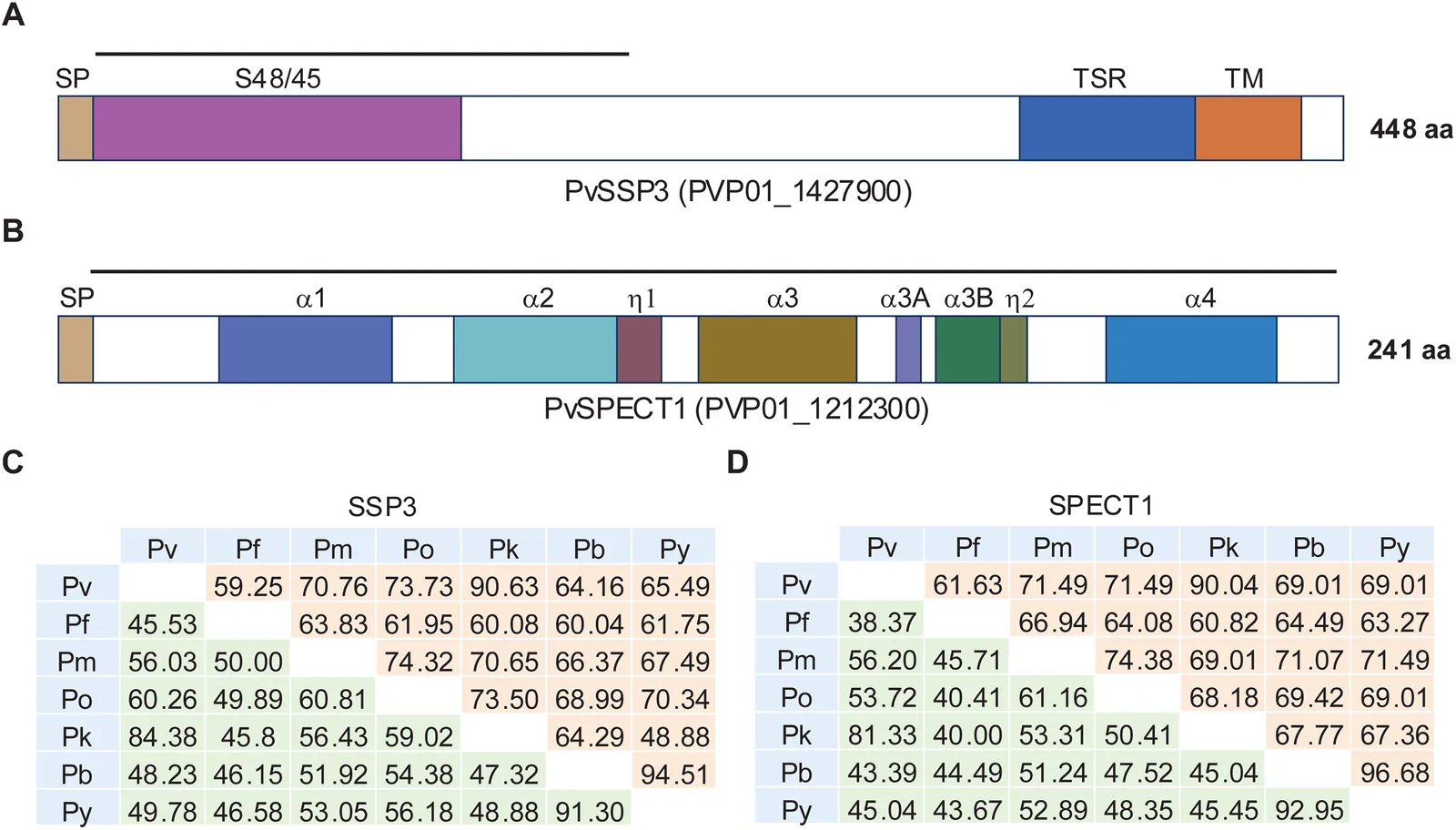
Features of antigens selected. **(A)** Schematic representation of *P. vivax* SSP3 protein showing different domains. SP - signal peptide, S48/45 - S48/45 domain of *Plasmodium* 6-Cys proteins, TSR - thrombospondin repeat, TM - transmembrane domain. The top line represents the region (AA 19-203) selected for recombinant *Pv*SSP3 production. **(B)** Schematic representation of *P. vivax* SPECT1 protein showing different domains. SP - signal peptide, α1, α2, α3 and α4 - four different alpha helices in the bundle, α3a and α3b - antiparallel α-helices in the hook structure. The black line at the top represents the region selected for recombinant *Pv*SPECT1 production. **(C and D)** Homology and Identity matrices of SSP3 and SPECT1 from different *Plasmodium* species were calculated using the sequence manipulation suite [61] **(C)** Homology and identity matrix of SSP3. Pv - *P. vivax* P01 (PVP01_1427900), Pf - *P. falciparum* 3D7 (PF3D7_0812300), Pm - *P. malariae* UG01 (PmUG01_14043900), Po - *P. ovale* curtisi GH01 (PocGH01_14035200), Pk - *P. knowlesi* strain H (PKNH_1428300), Pb - *P. berghei* ANKA (PBANKA_1425200), Py - *P. yoelii* 17X (PY17X_1427200). **(D)** Homology and identity matrix of SPECT1. Pv - *P. vivax* P01 (PVP01_1212300), Pf - *P. falciparum* 3D7 (PF3D7_1342500), Pm - *P. malariae* UG01 (PmUG01_12022400), Po - *P. ovale* curtisi GH01 (PocGH01_12020600), Pk - *P. knowlesi* strain H (PKNH_1258900), Pb - *P. berghei* ANKA (PBANKA_1355600), Py - *P. yoelii* 17X (PY17X_1361300).

#### *In vitro* EEF development.

To determine the ontogenic trajectory of transgenic parasites *in vitro*, parasite growth was assessed at different time points in the HC-04 human hepatoma cell line. HC-04 were seeded at 8x10^3^ cells/well in a collagen-coated 384-well plate (Greiner Bio-One) and maintained at 37°C in a CO_2_ incubator. The next day, HC-04 were infected with 2 × 10^3^ *Pb*WT or transgenic sporozoites expressing *P. vivax* proteins. The infected cultures were fixed at 12, 24, 36, 48, and 60 hours post-infection (hpi) with 4% paraformaldehyde (PFA, Thermo Fisher Scientific). In addition to this, at 48 hpi, *Pb*WT and *Pb-Pv*SSP3 expressing mCherry were stained with Hoechst 33342 (Thermo Fisher Scientific) for 30 min at 37°C, and the EEFs were imaged by live fluorescence using a high content imaging system, Cell Insight CX7 (Thermo Fisher Scientific). The parasite sizes and EEF numbers per well captured in JPEGs images were quantified using a custom in-house Python-based algorithm [23]. In the case of *Pb-Pv*SPECT1, infected wells were fixed at 48 hpi with 4% PFA. The parasite’s growth, size, and numbers were measured after staining the parasites by an indirect immunofluorescence assay.

#### Immunofluorescence assay (IFA).

Expression of the *Pv*SSP3 and *Pv*SPECT1 proteins in the transgenic sporozoites was revealed by an indirect immunofluorescence assay. The sporozoites were spotted on a Teflon-coated glass slide (Tekdon) and fixed with 4% PFA for 10–15 minutes, then washed with PBS. After blocking with 5% FBS for 1 hour at room temperature (RT), sporozoites were incubated overnight at 4°C at 1:500 dilution with mouse antisera generated using recombinant *Pv*SSP3 and *Pv*SPECT1 [7]. Pre-immune sera were used as negative controls (S1 Fig). The sporozoites were washed three times with PBS and incubated with goat anti-mouse secondary antibodies conjugated with either Alexa Fluor 488 or Alexa Fluor 594 at 1:1000 dilution and Hoechst 33342 at 1:1000 dilution for 1 hour at RT. The sporozoites were washed 3X with PBS, and the coverslip was mounted with Prolong Glass Antifade (Thermo Fisher Scientific). All the secondary antibodies used in the study were procured from Thermo Fisher Scientific.

To assess *in vitro* EEF development, the PFA fixed samples were incubated with a combination of mouse anti-*Pb*CSP monoclonal antibody (3D11) and goat anti-*Pb*UIS4 polyclonal antibody (LS Bio) or mouse anti-*Pb*MSP1 monoclonal (F4.4) and goat anti-*Pb*UIS4 polyclonal antibody at 1:1000 dilution in blocking buffer (1% BSA and 0.3% Triton X-100 in PBS) overnight at 4°C. The primary antibodies were removed, and wells were washed 3X with PBS and incubated with either a combination of Alexa Fluor 488 conjugated chicken anti-mouse antibodies and Alexa Fluor 594 conjugated donkey anti-goat antibodies or Alexa Fluor 594 conjugated chicken anti-mouse antibodies and Alexa Fluor 488 conjugated donkey anti-goat antibodies at 1:1000 dilution along with Hoechst 33342 at 1:1000 dilution in blocking buffer for 1 hour at RT. After incubation, the cells were washed three times with PBS, and fresh PBS was added to the wells. Images were acquired using Zen 3.0 Blue Edition on a Zeiss LSM-900 confocal microscope with Airyscan 2.

#### Inhibition of liver stage development assay (ILSDA).

Cultures of HC-04 as described above were seeded at 8x10^3^ per well. Sporozoites (50 spz/µl) were incubated for 20 min at room temperature in a 1:100 dilution of heat-inactivated mouse serum of pre-immune, adjuvant only (TitreMax Gold), or sera against r*Pv*SSP3 or r*Pv*SPECT1, generated in the previous study [7]. In six replicate wells per serum, 2x10^3^ sporozoites were added per well, and the plates were centrifuged at 200 × g for 5 min. The plates were incubated at 37°C in a CO_2_ incubator to facilitate hepatocyte invasion. The extracellular sporozoites were removed by a medium exchange at 1 hpi, followed by an additional medium replacement at 24 hpi. The nuclei of the liver stages of *Pb-Pv*SSP3 expressing mCherry were stained with Hoechst 33342 at 48 hpi for 30 min at 37°C, and the EEFs were imaged by live fluorescence using Cell Insight CX7. The liver stages of *Pb-Pv*SPECT1 transgenic line were fixed at 48 hpi with 4% PFA. To determine the EEF numbers and size of *Pb-Pv*SPECT1, parasites were stained with goat anti-*Pb*UIS4 polyclonal antibody at 1:1000 dilution in blocking buffer and incubated overnight at 4°C. The wells were washed 3X with PBS and incubated with Alexa Fluor 594 conjugated donkey anti-goat antibodies at a 1:1000 dilution, along with Hoechst 33342 at a 1:1000 dilution for 1 hour at RT. After incubation, the cells were washed 3X with PBS, and fresh PBS was added to the wells. Images were acquired with the Cell Insight CX7 high-content imaging system. The images were exported as JPEGs, and parasite sizes and the number of EEFs per well were calculated from the JPEG images using an in-house Python program [23]. The percent inhibition for each well was calculated using the formula: “Percent inhibition of infection = [1 - (Mean EEFs in test serum/Mean EEFs in pre-immune serum)] × 100”. Three independent ILSDA assays were performed for each serum, and the average percent inhibition was calculated. EEF numbers per well and EEF sizes were quantified using data from at least eight replicate wells across four independent experiments.

#### *In vivo* sporozoite challenge.

Sporozoite challenge experiments were performed with 3 groups of 6–8 weeks old female BALB/c mice for each antigen: antigen-immunized (n = 10), adjuvant control (n = 10), and naïve (n = 5). Mice were immunized twice subcutaneously with 25 µg of either r*Pv*SSP3 or r*Pv*SPECT1, emulsified in TitreMax Gold (CytRx Corporation), on Days 0 and 21. Mice immunized with TitreMax Gold/PBS (1:1) served as controls. On day 42, the mice were challenged with 500 transgenic *P. berghei* sporozoites expressing either *Pv*SSP3 or *Pv*SPECT1 by intravenous injection. Mice were monitored for breakthrough blood-stage infection from day 5 post-challenge by examining Giemsa-stained tail blood smears. Mice were considered patent when parasitemia reached 1% counted against at least 10,000 RBCs in Giemsa-stained tail blood smears.

#### Enzyme-Linked immunosorbent assay (ELISA).

On day 42 after immunization, serum antibody titers in BALB/c mice immunized with r*Pv*SSP3 or r*Pv*SPECT1 formulated in TitreMax Gold were measured by endpoint ELISA. Briefly, 96-well microtiter plates (NUNC, USA) were coated overnight at 4°C with 3 µg/ml of either r*Pv*SSP3 or r*Pv*SPECT1 in 1X PBS (100 µl/well). The plates were washed with PBS containing 0.05% Tween 20 (PBST) and blocked with 5% nonfat milk in PBST for 2 h at room temperature (RT). Immune sera were serially diluted threefold, beginning at 1:100, in 2% milk/PBST and incubated in duplicate wells for 2 h at RT. After three washes with PBST, the plates were incubated with 100 µl of 0.5 µg/ml alkaline phosphatase-conjugated goat anti-mouse secondary antibody (SeraCare Life Sciences) for 90 min at RT. The plates were then washed three times with PBST, and bound total IgG was detected using 100 µl of alkaline phosphatase substrate (BluePhos Microwell Substrate Kit; SeraCare Life Sciences). Absorbance was measured at 650 nm using an automated microplate reader (CLARIOstar Plus, BMG Labtech).

### Statistical analysis

GraphPad Prism v11 was used for statistical analysis. Normality was assessed for all data, except pre-patency data, using the Shapiro-Wilk test before analysis. Statistical analyses were performed using the Kruskal-Wallis test for oocyst numbers per mosquito and for measuring the parasite liver loads (RLUs), one-way ANOVA with Tukey’s multiple comparisons test for sporozoite numbers per mosquito, EEF numbers, and sizes, and One-way ANOVA with Holm-Sidak’s multiple comparisons test for ILSDAs. Mantel-Cox tests were used to assess differences in pre-patency periods.

## Results

### Selection of antigens

Advanced omics studies of *Plasmodium* sporozoites have identified several surface proteins that could serve as potent subunit vaccine antigens [30–33]. We selected SSP3 and SPECT1 because their upregulation in activated sporozoites correlated with infectivity and their putative roles in the PE infection cascade [32]. SSP3 is essential for gliding motility in *P. yoelii,* and in *P. berghei*, it is essential for EEF maturation, whereas SPECT1 plays a key role in sporozoite migration through host cells, such as Küpffer cells and the liver sinusoidal wall, and both play critical roles in completing the liver-stage infection [28,34,35]. SSP3 has an S48/45 domain at the N-terminus and a thrombospondin type I repeat (TSR) domain at the C-terminus, just before the transmembrane domain (Fig 1A) [34]. The 13 cysteines of SSP3 are conserved across *Plasmodium* species. SPECT1 is a micronemal protein that has 4 alpha helices, α1 to α4 (Fig 1B). It undergoes a conformational change to facilitate pore formation in host cell membranes [36]. *Pv*SSP3 shares 48% identity and 64% homology with *P. berghei* SSP3 (Fig 1C), whereas *Pv*SPECT1 shares 43% identity and 69% homology with *P. berghei* SPECT1 (Fig 1D). Preclinical studies showed that mouse antisera raised against these antigens can significantly inhibit sporozoite invasion and attenuate *P. vivax* liver-stage development *in vitro* [7]. In malaria-endemic regions, naturally exposed residents exhibited neutralizing antibodies against *Pv*SSP3 and *Pv*SPECT1 [23].

### Generation of chimeric *P. berghei* expressing *P. vivax* SSP3 and SPECT1

To evaluate the immune efficacy of *P. vivax* pre-erythrocytic antigens SSP3 and SPECT1, transgenic *P. berghei* chimeric lines expressing *P. vivax* SSP3 and SPECT1 were generated using a two-step gene insertion/marker out (GIMO) based transfection technology [43]. A chimeric *Pb-Pv*SSP3 transgenic line was successfully generated using the parental *P*. *berghei* ANKA parasite line 1868cl1 that constitutively expresses mCherry luciferase reporter, hereafter referred to as *Pb*WT (Fig 2A). In this line, the gene encoding *Pbssp3* (PBANKA_1425200) was replaced in two steps with *Pvssp3* (PVP01_1427900) coding sequence (Fig 2B), resulting in *Pb-Pv*SSP3 (line 3395cl1). In the *Pb-Pv*SSP3 line, the *Pvssp3* gene is under the control of the *Pbssp3* gene promoter and the transcription terminator. Correct integration of the targeting constructs in both transfection steps was confirmed by diagnostic Southern blot hybridization analysis of chromosomes separated by pulsed-field gel electrophoresis and by diagnostic PCR (Fig 2C and 2D). Chimeric *Pb-Pv*SPECT1 transgenic line was generated using the parental *P*. *berghei* ANKA parasite line 676m1cl1, which constitutively expresses a fused GFP*-*luciferase reporter (Fig 3A). In this line, the gene encoding *Pbspect1* (PBANKA_1355600) was replaced in two steps with *Pvspect1* (PVP01_1212300) coding sequence (Fig 3B), resulting in *Pb-Pv*SPECT1 (line 3378cl1). In the *Pb-Pv*SPECT1 line, the *Pvspect1* gene is under the control of the *Pbspect1* gene promoter and the transcription terminator. Correct integration of the targeting constructs was confirmed by diagnostic Southern blot hybridization analysis of chromosomes separated by pulsed-field gel electrophoresis and by diagnostic PCR (Fig 3C and 3D).

**Fig 2:**
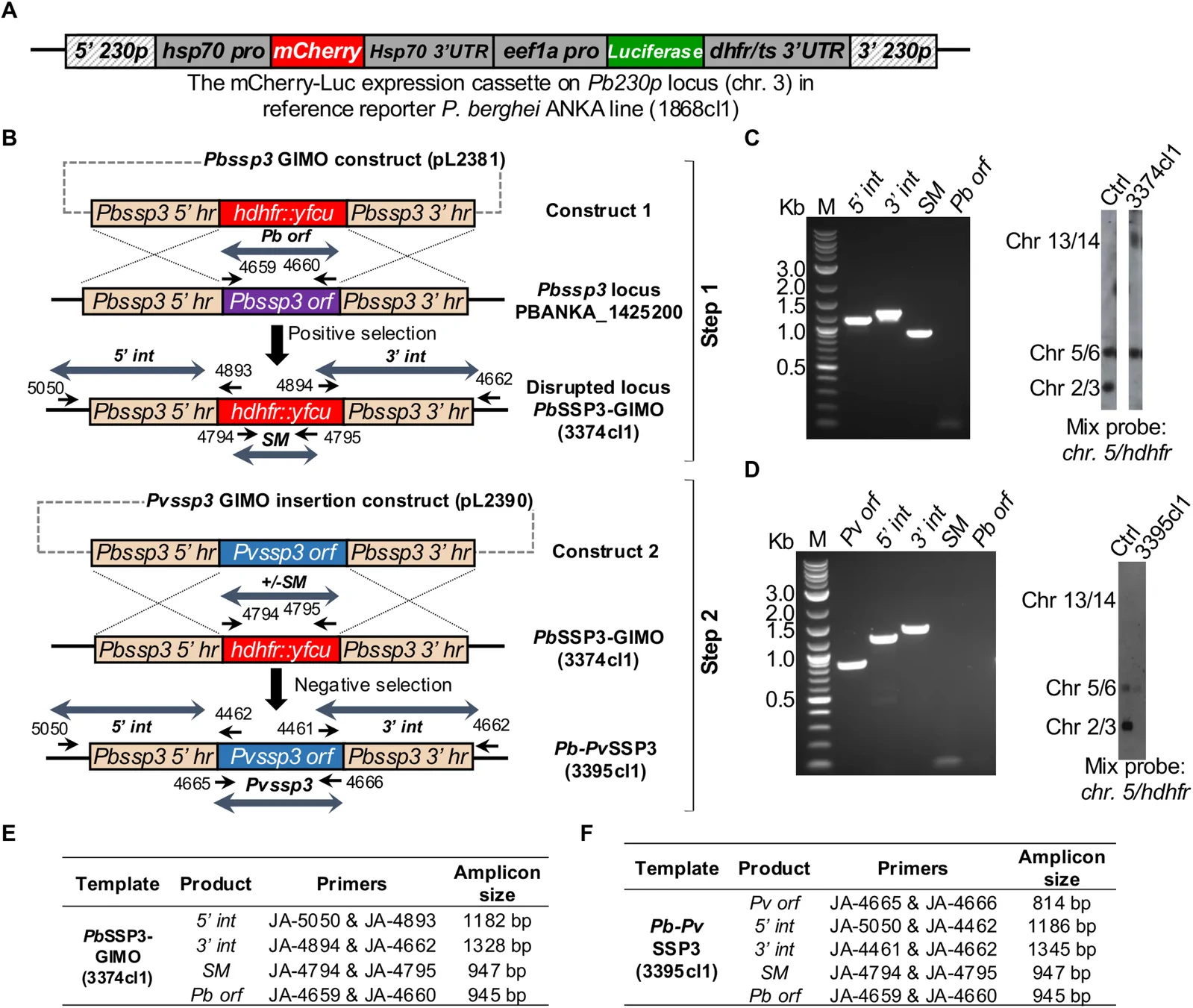
Generation and genotyping of *P. berghei* transgenic parasite line expressing *Pv*SSP3. **(A)** *P. berghei* ANKA reference reporter line (1868cl1) showing *mCherry* and *luciferase* reporter genes under the constitutive *hsp70* and *eef1a*, respectively, introduced at the neutral *230p* locus. This reporter line was used to generate the *Pb-Pv*SSP3 replacement line. **(B)** Schematic representation of the generation of *P. vivax* SSP3 replacement line *Pb-Pv*SSP3 (line 3395cl1). Step 1: *Pbssp3 open reading frame* (*orf*) was replaced with h*dhfr*::*yfcu* positive-negative selectable marker cassette of *Pbssp3* GIMO construct (Construct 1, pL2381) to generate *Pb*SSP3*-*GIMO (line 3374) by double crossover homologous recombination applying positive selection with pyrimethamine. After genotyping and confirmation of correct construct integration, this line was cloned by limiting dilution, and cl1 (3374cl1) was selected for Step 2. Int, integration. Step 2: The GIMO insertion construct (construct 2; pL2390) was used to replace the SM in the *Pb*SSP3*-*GIMO line with the *Pvssp3 orf*, resulting in the generation of marker free line *Pb-Pv*SSP3 (line 3395cl1) after negative (5-FC) selection. Construct 2 integrates by double cross-over homologous recombination using the same targeting regions employed in construct 1, resulting in the introduction of the *Pvssp3 orf* under the control of *Pbssp3* regulatory sequences. Black arrows: number and location of primers used for diagnostic PCR (panels **C** and **D**). **(C)** Left panel: Genotype analysis of *Pb*SSP3-GIMO parasites by diagnostic PCR confirms the absence of *Pbssp3 orf*, the correct integration of the constructs at both the 5’ and 3’ regions (*5’* int and *3’* int), and the presence of the h*dhfr*::y*fcu* SM. Primer sequences are shown in S1 Table and expected PCR fragment sizes and primer names are listed in **E**. Right panel: Genotype analysis of *Pb*SSP3*-*GIMO parasites by Southern analysis of chromosomes (chr.) separated by pulsed-field gel electrophoresis (PFGE). Hybridization of PFGE-separated chr. of *Pbssp3* with a mixture of a h*dhfr* probe and a probe specific for chr. 5 confirms the integration of pL2381 into the *Pb*SSP3-GIMO locus on chr. 14. As an additional control (ctrl), parasite line 2117cl1 is used with the h*dhfr*::y*fcu* SM integrated into chr. 3. **(D)** Left panel: Genotype analysis of *Pb-Pv*SSP3 parasites by diagnostic PCR confirms the presence of *Pvssp3 orf*, the correct integration of the constructs at both the 5’ and 3’ regions (*5’* int and *3’* int), and the absence of the h*dhfr*::y*fcu* SM. Primer sequences are shown in S1 Table and expected PCR fragment sizes and primer names are listed in **F**. Right panel: Genotype analysis of *Pb-Pv*SSP3 parasites by Southern analysis of chromosomes (chr.) separated by pulsed-field gel electrophoresis (PFGE). The correct integration of construct 2 in *Pb-Pvssp3* (line 3395) was confirmed by showing the absence of the h*dhfr*::y*fcu* selectable marker (SM) cassette in cloned chimeric parasites by hybridization of chr. with the *hdhfr* probe. Chromosomes are also hybridized to a control probe recognizing chr. 5. As an additional control (ctrl), parasite line 2117cl1 is used with the h*dhfr*::y*fcu* SM integrated into chr. 3. **(E and F)** Expected fragment sizes and primer names used for diagnostic PCR of *Pb*SSP3-GIMO (**E**) and *Pb-Pv*SSP3 **(F)**.

**Fig 3:**
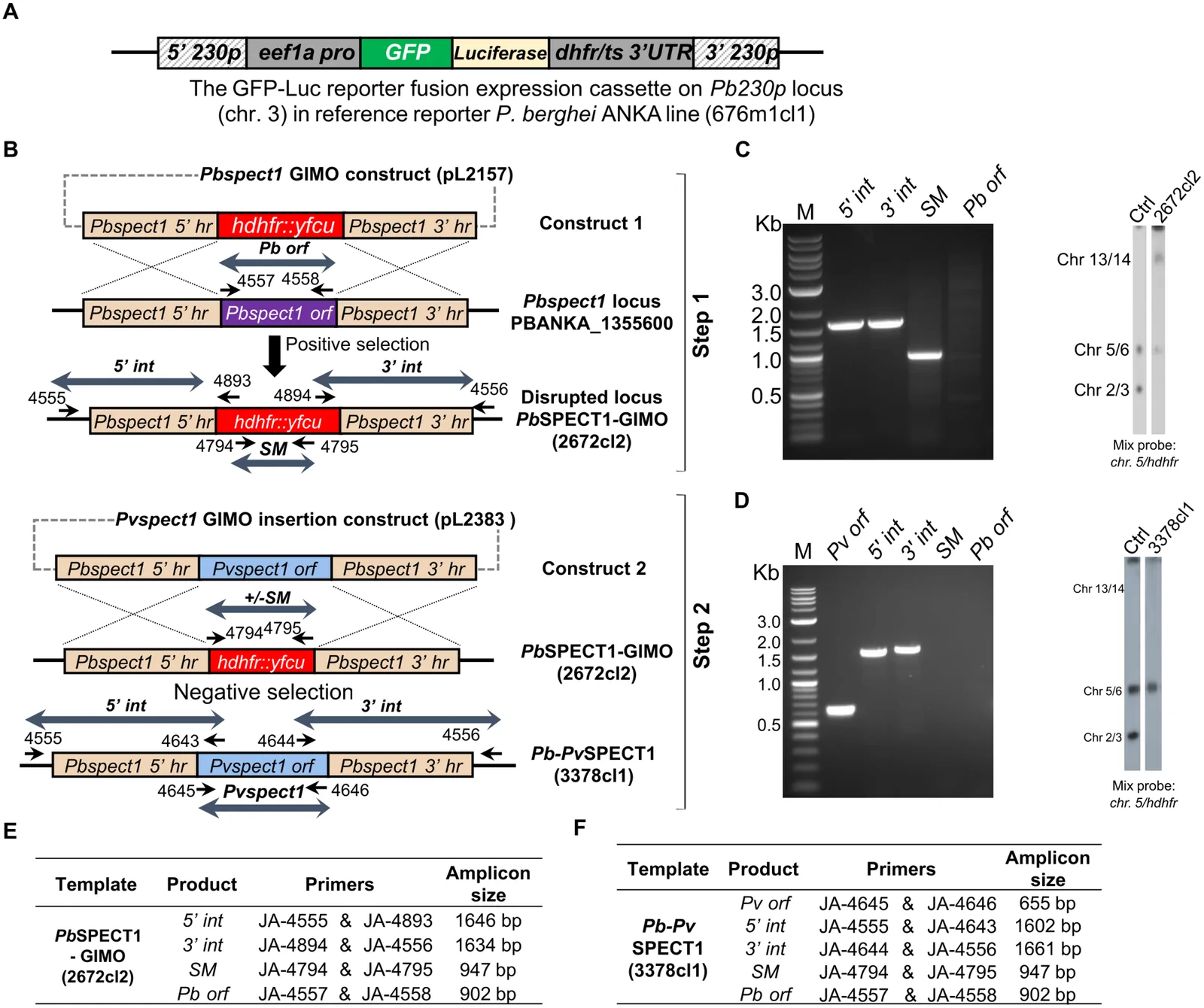
Generation and genotyping of *P. berghei* line transgenic parasite line expressing *Pv*SPECT1. **(A)** *P. berghei* ANKA reference reporter line (676m1cl1) showing *gfp* fused with the *luciferase* reporter gene under the constitutive *eef1a* promoter introduced at the neutral *230p* locus. This reporter line was used to generate the *Pb-Pv*SPECT1 replacement line. **(B)** Schematic representation of the generation of *P. vivax* SPECT1 replacement line *Pb-Pv*SPECT1 (line 3378cl1). Step 1: The *Pbspect1 orf* was replaced with h*dhfr*::*yfcu* positive-negative selectable marker cassette of *Pbspect1* GIMO construct (Construct 1, pL2157) to generate *Pb*SPECT1*-*GIMO (line 2672) by double crossover homologous recombination, applying positive selection with pyrimethamine. After genotyping and confirmation of correct construct integration, this line was cloned by limiting dilution, and cl2 (2672cl2) was selected for Step 2. Int, integration. Step 2: The *Pvspect1* GIMO insertion construct (pL2383) was used to replace the h*dhfr*::y*fcu* SM in the *Pb*SPECT1-GIMO line with the *Pvspect1 orf*, resulting in the generation of line *Pb-Pv*SPECT1 (line 3378) after negative (5-FC) selection. Construct pL2383 integrates by double-crossover homologous recombination using engineered *Pbspect1* 5’ and 3’ homology regions, introducing the *Pvspect1 orf* under the control of *Pbspect1* regulatory sequences. Black arrows: number and location of primers used for diagnostic PCR (panels **C** and **D**). **(C)** Left panel: Genotype analysis of *Pb*SPECT1-GIMO parasites by diagnostic PCR confirms the absence of *Pbspect1 orf*, the correct integration of the constructs at both the 5’ and 3’ regions (*5’* int and *3’* int), and the presence of the h*dhfr*::y*fcu* SM. Primer sequences are shown in S1 Table and expected PCR fragment sizes and primer names are listed in **E**. Right panel: Genotype analysis of *Pb*SPECT1*-*GIMO parasites by Southern analysis of chromosomes (chr.) separated by pulsed-field gel electrophoresis (PFGE). Hybridization of PFGE-separated chr. of *Pbspect1* with a mixture of a h*dhfr* probe and a probe specific for chr. 5 confirms the integration of pL2157 into the *Pb*SPECT1-GIMO locus on chr. 13. As an additional control (ctrl), parasite line 2117cl1 is used with the h*dhfr*::y*fcu* SM integrated into chr. 3. **(D)** Left panel: Genotype analysis of *Pb-Pv*SPECT1 parasites by diagnostic PCR confirms the presence of *Pvspect1 orf*, the correct integration of the constructs at both the 5’ and 3’ regions (*5’* int and *3’* int), and the absence of the h*dhfr*::y*fcu* SM. Primer sequences are shown in S1 Table, and expected PCR fragment sizes and primer names are listed in panel **F**. Right panel: Genotype analysis of *Pb-Pv*SPECT1 parasites by Southern analysis of chromosomes (chr.) separated by pulsed-field gel electrophoresis (PFGE). The correct integration of *Pvspect1* GIMO insertion construct in *Pb-Pv*SPECT1 (line 3378) was confirmed by showing the absence of the h*dhfr*::y*fcu* selectable marker (SM) cassette in cloned chimeric parasites by hybridization of chr. with the *hdhfr* probe. Chromosomes are also hybridized to a control probe recognizing chr. 5. As an additional control (ctrl), parasite line 2117cl1 is used with the h*dhfr*::y*fcu* SM integrated into chr. 3. **(E and F)** Expected fragment sizes and primer names used for diagnostic PCR of *Pb*SPECT1-GIMO (**E**) and *Pb-Pv*SPECT1 **(F)**.

### *Pb-Pv*SSP3 and *Pb-Pv*SPECT1 transgenic lines show WT-like growth in mice and mosquitoes

To evaluate the impact of gene replacement in *P. berghei* transgenic parasites, the complete life cycle was systematically examined in both the mouse and the mosquito vector. In this regard, the *in vivo* propagation of asexual blood stages was first determined during the cloning procedure for the *Pb-Pv*SSP3 and *Pb-Pv*SPECT1 transgenic lines [46]. Wild-type *P. berghei* (n = 10) showed a parasitemia of 0.5% - 2% on day 8 after injection of a single parasite. We obtained five *Pb-Pv*SPECT1 clones and three *Pb-Pv*SSP3 clones, all of which showed a parasitemia ranging from 0.5% to 2% on day 8 after injection of a single parasite, indicating that the blood stage asexual multiplication rate of *Pb-Pv*SSP3 and *Pb-Pv*SPECT1 transgenic lines was comparable to wild type *P. berghei* ANKA parasites. Following transmission to mosquitoes, midgut oocysts were quantified on day 14 post-infection, and salivary gland sporozoite numbers on days 18–21 post infection. Both *Pb-Pv*SSP3 and *Pb-Pv*SPECT1 produced a similar number of oocysts in the mosquito midguts as that of *Pb*WT, with no statistically significant difference between groups (*p* > 0.99 for both lines, Kruskal-Wallis test with Dunn’s multiple comparison test) (Fig 4A), and the salivary gland sporozoite numbers were comparable to those of *Pb*WT parasites (*p* = 0.98 for *Pb-Pv*SSP3 and *p* = 0.99 for *Pb-Pv*SPECT1, One-Way ANOVA with Tukey’s multiple comparison test) (Fig 4B).

**Fig 4:**
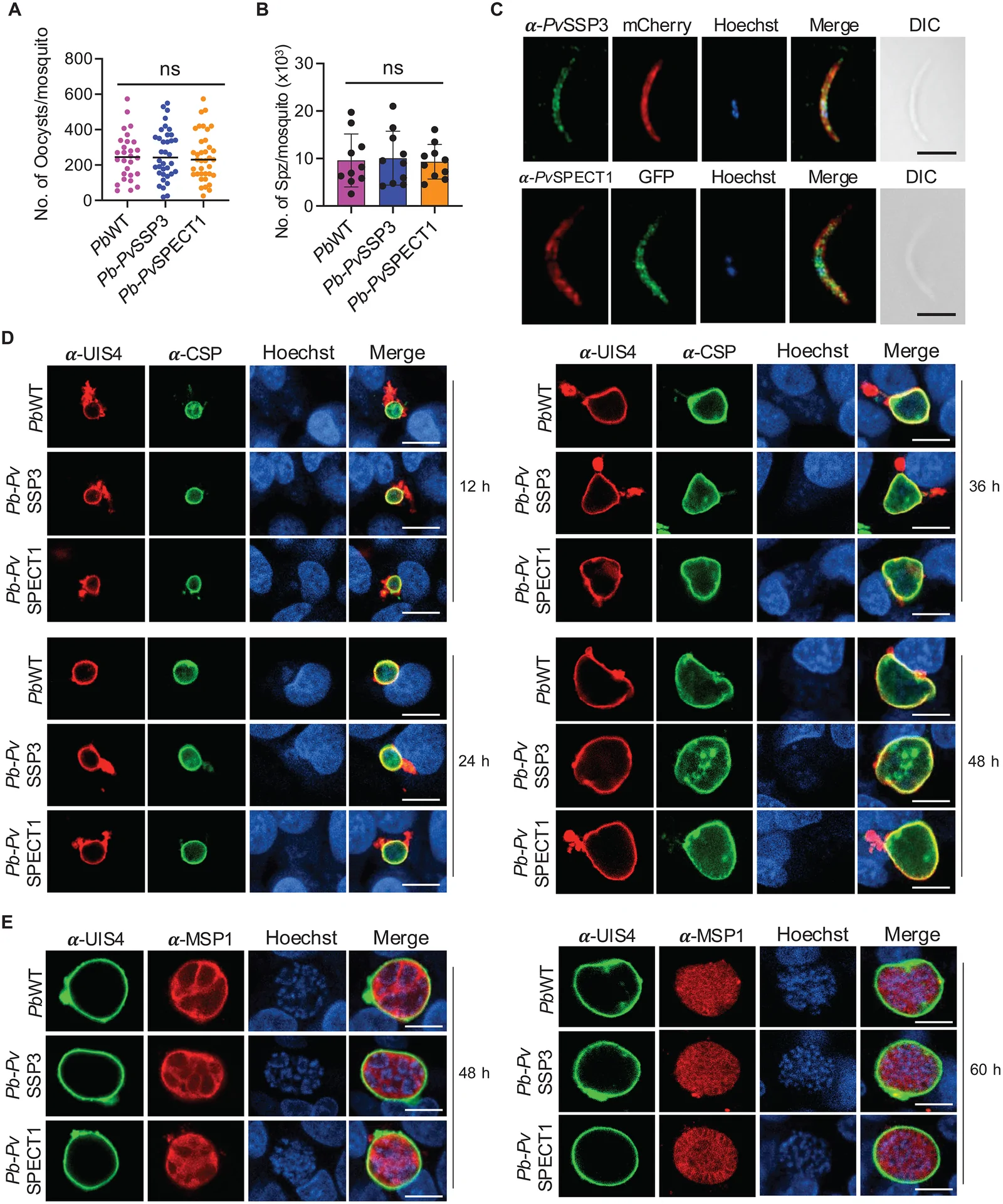
Fitness analysis of *Pb-Pv*SSP3 and *Pb-Pv*SPECT1 line in mosquitoes and *in vitro* liver stages: **(A)** Dot plot showing the number of oocysts per mosquito in *A. stephensi* mosquitoes infected with *Pb* wild type (*Pb*WT), *Pb-Pv*SSP3, and *Pb-Pv*SPECT1. Each dot represents the number of oocysts from an individual mosquito. Data are pooled from three independent feeding experiments, with 8-15 mosquitoes analyzed per experiment. Horizontal line showing Median. n = 28 for *Pb*WT, 36 for *Pb-Pv*SSP3, and 37 for *Pb-Pv*SPECT1. The Shapiro-Wilk test for normality confirmed that oocyst numbers were normally distributed. Significance was assessed using the Kruskal-Wallis test (*p* > 0.99), ns - not significant. **(B)** Bar graph showing the salivary gland sporozoite loads per mosquito in *A. stephensi* mosquitoes infected with *Pb*WT, *Pb-Pv*SSP3, and *Pb-Pv*SPECT1. Each bar represents mean±SD. n = 10, with at least 60 mosquitoes per feeding experiment. The Shapiro-Wilk test for normality confirmed that the sporozoite numbers were normally distributed. Therefore, significance was calculated using One-way ANOVA with Tukey’s multiple comparisons test (*p* = 0.98 for *Pb-Pv*SSP3 and *p* = 0.99 for *Pb-Pv*SPECT1), ns - not significant. **(C)** Indirect immunofluorescence analysis of *Pb-Pv*SSP3 and *Pb-Pv*SPECT1 sporozoites showing the localization of *Pv*SSP3 and *Pv*SPECT1 on sporozoites. *Pb-Pv*SSP3 sporozoites were stained with anti-*Pv*SSP3 polyclonal mice serum and revealed with Alexa Fluor 488 conjugated anti-mouse secondary antibody (top panel). *Pb-Pv*SPECT1 sporozoites were stained with anti-*Pv*SPECT1 polyclonal mice serum and revealed with Alexa Fluor 594 conjugated anti-mouse secondary antibody (bottom panel). Nuclei were stained with Hoechst 33342. Scale bar - 5 μm. **(D)** EEF development of *Pb-Pv*SSP3 and *Pb-Pv*SPECT1 mutants in HC-04 cells stained with anti-CSP and anti-UIS4 antibodies at the indicated time points. The *in vitro* EEFs fixed at 12 h, 24 h, 36 h, and 48 h were stained with mouse anti-CSP monoclonal antibody (3D11), and the parasitophorous vacuolar membrane (PVM) was stained with goat anti-UIS4 polyclonal antibody. Immunoreactivity was revealed with Alexa Fluor 488 conjugated donkey anti-mouse secondary antibody and Alexa Fluor 594 conjugated donkey anti-goat secondary antibody. The host and parasite nuclei were stained with Hoechst 33342 (Scale bar - 10 μm). **(E)** EEF development of *Pb-Pv*SSP3 and *Pb-Pv*SPECT1 mutants in HC-04 cells stained with MSP1 and UIS4. The *in vitro* EEFs fixed at 48 h and 60 h were stained with mouse anti-*Pb*MSP1 monoclonal antibody (F4.4), and the parasitophorous vacuolar membrane (PVM) was stained with goat anti-UIS4 antibody. Immunoreactivity was revealed with Alexa Fluor 594 conjugated donkey anti-mouse secondary antibody and Alexa Fluor 488 conjugated donkey anti-goat secondary antibody. The host and parasite nuclei were stained with Hoechst 33342 (Scale bar - 10 μm).

### Antisera to *Pv*SSP3 and *Pv*SPECT1 are reactive with native SSP3 and SPECT1 in Sporozoites

We next assessed expression of *Pv*SSP3 and *Pv*SPECT1 in the transgenic *Pb-Pv*SSP3 and *Pb-Pv*SPECT1 lines using antisera raised in mice against the corresponding recombinant proteins [7]. Immunofluorescence analysis revealed clear surface staining of sporozoites from both transgenic lines, confirming expression of *Pv*SSP3 in *Pb-Pv*SSP3 sporozoites (Fig 4C, top panel) and *Pv*SPECT1 in *Pb-Pv*SPECT1 sporozoites (Fig 4C, bottom panel). By contrast, no immunoreactivity was detected with pre-immune serum, supporting the specificity of the antisera (S1 Fig).

### *Pb-Pv*SSP3 and *Pb-Pv*SPECT1 parasites show normal liver stage development

To assess the liver stage development of *Pb-Pv*SSP3 and *Pb-Pv*SPECT1 chimeric parasites, 2x10^3^ sporozoites were added to the HC-04 monolayers, and the EEF development was monitored at 12, 24, 36, and 48 hours post infection by IFA with anti-CSP and anti-UIS4 and at 48 and 60 hours post infection by staining with anti-MSP1 and anti-UIS4. The growth of *Pb-Pv*SSP3 and *Pb-Pv*SPECT1 transgenic EEFs is visually comparable to that of *Pb*WT at all time points, and a representative image at each time point is shown in Fig 4D and 4E. Additionally, the number and sizes of the EEFs were quantified at 48 hours post infection. The number of EEFs (Fig 5A, *p* = 0.88 for *Pb- Pv*SSP3 and *p* = 0.99 for *Pb-Pv*SPECT1) and the size of EEFs (Fig 5B, *p* = 0.89 for *Pb-Pv*SSP3 and *p* = 0.97 for *Pb-Pv*SPECT1) were also not significantly different from those of *Pb*WT (One-Way ANOVA with Tukey’s multiple comparison test). We further analyzed the infectivity of the transgenic sporozoites to mice by intravenous injection of 1 x 10^4^ *Pb*WT or transgenic sporozoites into mice. At 44 h post infection, the parasite liver burden was measured by *in vivo* imaging. The RLU (Relative Light Units) of *Pb-Pv*SSP3 and *Pb-Pv*SPECT1 parasites are comparable to those of *Pb*WT, with no significant differences (*p* = 0.68 for *Pb-Pv*SSP3 and *p* = 0.99 for *Pb-Pv*SPECT1, One-Way ANOVA with Tukey’s multiple comparison test) (Figs 5C and S2). The pre-patency period was analyzed by counting parasites in Giemsa-stained tail blood smears. *Pb-Pv*SSP3 and *Pb-Pv*SPECT1 parasite lines showed a median pre-patency of day 5, similar to that of *Pb*WT, with no significant difference (Log-rank (Mantel-Cox) test, *p =* 0.94) (Fig 5D).

**Fig 5:**
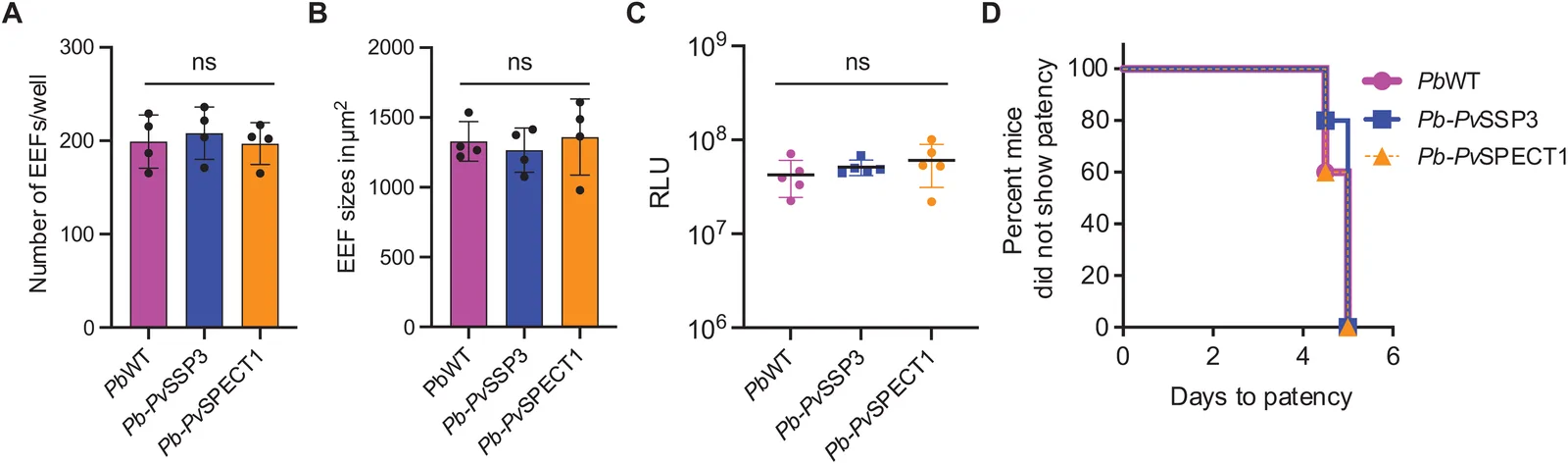
*In vitro* and *in vivo* infectivity of *Pb-Pv*SSP3 and *Pb-Pv*SPECT1 sporozoites. (**A**) Bar graph showing the number of EEFs per well at 48 hours post infection (hpi) in an HC-04 monolayer infected with 2 x 10^3^ of *Pb*WT, *Pb-Pv*SSP3 or *Pb-Pv*SPECT1 sporozoites in a 384-well plate. Data were obtained from 4 independent experiments, each with at least 8 wells. Error bars represent the mean with the standard deviation. A Shapiro-Wilk test confirmed that the EEF numbers/well were normally distributed. Significance was calculated using One-way ANOVA with Tukey’s multiple comparisons test (*p* = 0.88 for *Pb-Pv*SSP3 and *p* = 0.99 for *Pb-Pv*SPECT1), ns - not significant. (**B**) Bar graph showing the average size of EEF in µm^2^ at 48 hpi in an HC-04 monolayer infected with *Pb*WT, *Pb-Pv*SSP3 or *Pb-Pv*SPECT1 sporozoites in a 384-well plate. Data are obtained from 4 independent experiments, each with at least 1000 EEFs. Error bars represent the mean with the standard deviation. A Shapiro-Wilk test confirmed that the EEF sizes were normally distributed. Significance was calculated using One-way ANOVA with Tukey’s multiple comparisons test (*p* = 0.89 for *Pb-Pv*SSP3 and *p* = 0.97 for *Pb-Pv*SPECT1), ns - not significant. (**C**) Dot plot showing the infectivity of *Pb*WT, *Pb-Pv*SSP3 or *Pb-Pv*SPECT1 sporozoites as determined by parasite liver loads after intravenous injection of mice (n = 5) with 1x10^4^ sporozoites. Parasite liver loads were determined at 44 h by measuring *in vivo* luciferase activity and depicted as relative light units (RLU). Error bars represent the mean with the standard deviation. The Shapiro-Wilk test indicated that the assumption of normality was violated in the *Pb-Pv*SSP3 group (*p* < 0.05). Hence, significance was calculated using a nonparametric Kruskal-Wallis test (*p* = 0.68 for *Pb-Pv*SSP3 and *p* = 0.99 for *Pb-Pv*SPECT1) ns - not significant. (**D**) Kaplan-Meier curve showing the prepatent period of *Pb*WT, *Pb-Pv*SSP3, and *Pb-Pv*SPECT1 sporozoites in Swiss ND4 mice (n = 5). Pre-patency is defined as the time to 1% parasitemia. Significance was calculated using the Log-Rank (Mantel-Cox) test (*p =* 0.94), no significant difference was observed between the three groups.

### SSP3 and SPECT1 antisera block hepatocyte invasion by transgenic sporozoites

The protective efficacy of the immune sera generated in mice using the recombinant *Pv*SSP3 and *Pv*SPECT1 [7] was assessed by ILSDA. The assay was performed in a 384-well format using HC-04 cell monolayers infected with transgenic *Pb-Pv*SSP3 and *Pb-Pv*SPECT1 sporozoites [4,23]. Although the magnitude of inhibition varied across individual sera, immune sera significantly reduced sporozoite infection of hepatocytes compared with TitreMax Gold adjuvant control (One-way ANOVA with Holm-Sidak’s multiple comparisons test, *p* = 0.0010 for *Pv*SSP3 and *p* = 0.0101 for *Pv*SPECT1) and the no-antibody control (One-way ANOVA with Holm-Sidak’s multiple comparisons test, *p* = 0.0003 for *Pv*SSP3 and *p* = 0.0101 for *Pv*SPECT1). The inhibitory activity of *Pv*SSP3 antiserum ranged from 16% to 43%, with a mean inhibition of 27% (SD = 9.8%) (Fig 6A). The inhibitory activity of *Pv*SPECT1 antiserum ranged from 8% to 27%, with a mean inhibition of 14% (SD = 7.5%) (Fig 6B).

**Fig 6:**
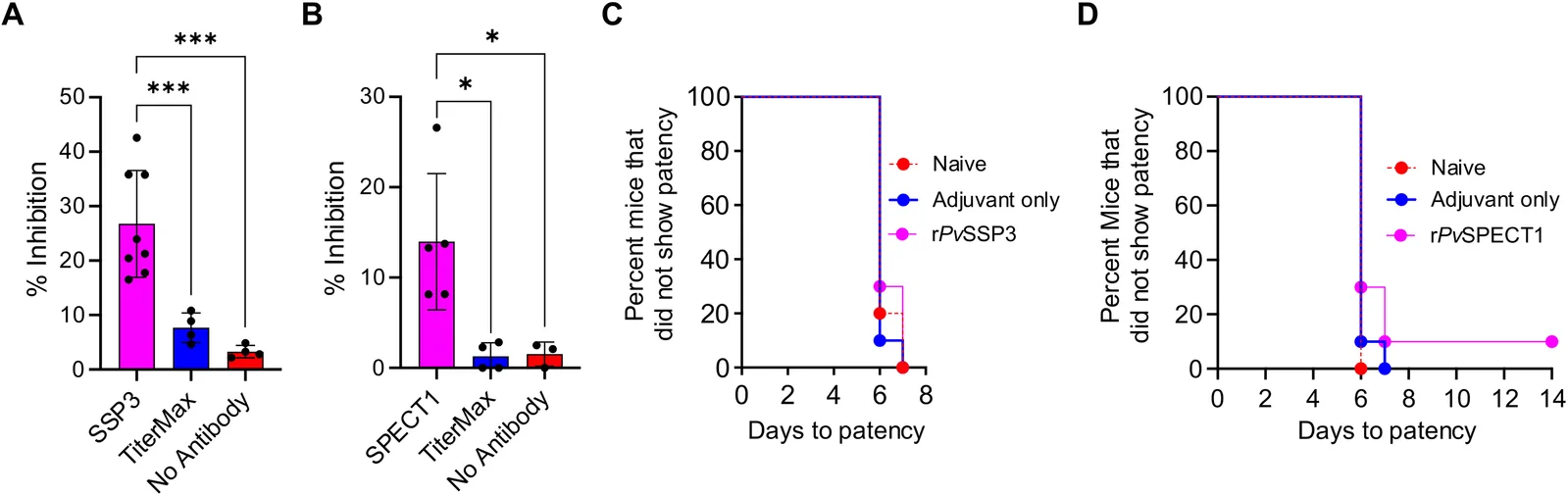
Antisera against *Pv*SSP3 and *Pv*SPECT1 inhibit hepatocyte invasion, and *Pv*SPECT1 confers limited protection in BALB/c mice after sporozoite challenge. (**A** and **B**) *In vitro* inhibition of liver-stage development assay (ILSDA) using 2 x 10^3^ transgenic *P. berghei* sporozoites expressing *Pv*SSP3 (*Pb-Pv*SSP3) or *Pv*SPECT1 (*Pb-Pv*SPECT1). Immune sera were generated by immunizing BALB/c mice with recombinant *Pv*SSP3 or *Pv*SPECT1 adjuvanted with Titremax Gold [7]. *Pb-Pv*SSP3 or *Pb-Pv*SPECT1 sporozoites were incubated with 1:100 dilution of the immune sera for 20 min at room temperature and added to monolayers of HC-04 line in 384-well plates for infection. Reduction in infection was expressed as a percentage inhibition of EEF formation by the immune sera relative to pre-immune serum. (**A**) Bar graph showing the percent inhibition of *Pb-Pv*SSP3 EEFs by *Pv*SSP3 immune serum. Each dot represents the serum from an individual mouse (n = 8), and the data were obtained from 4 independent experiments, each containing either triplicate or 6 replicate wells. A Shapiro-Wilk test confirmed that the inhibition data were normally distributed. Significance was determined by One-way ANOVA with Holm-Sidak’s multiple comparisons test (*p* = 0.0010 for *Pv*SSP3 vs TitreMax and *p* = 0.0003 for *Pv*SSP3 vs No Antibody). (**B**) Bar graph showing the percent inhibition of *Pb-Pv*SPECT1 EEFs by *Pv*SPECT1 immune serum. Each dot represents the serum from an individual mouse (n = 5), and the data were obtained from 4 independent experiments, each containing either triplicate or 6 replicate wells. A Shapiro-Wilk test confirmed that the inhibition data were normally distributed. Significance was determined by One-way ANOVA with Holm-Sidak’s multiple comparisons test (* *p* = 0.0101). (**C** and **D**) Naïve (n = 5), TitreMax Gold adjuvant (n = 10), or recombinant *Pv*SSP3 or *Pv*SPECT1 vaccinated mice (n = 10) were challenged with 500 sporozoites of either *Pb-Pv*SSP3 or *Pb-Pv*SPECT1. The pre-patency periods in mice were determined from Giemsa-stained tail blood smears. Prepatency is defined as the time to 1% parasitemia. (**C**) Kaplan-Meier curve showing the prepatent period of *Pb-Pv*SSP3 sporozoites. Significance was calculated using the Log-Rank (Mantel-Cox) test (*p* = 0.9), and no significant difference was observed between the three groups. (**D**) Kaplan-Meier curve showing the prepatent period of *Pb-Pv*SPECT1 sporozoites. Significance was calculated using the Log-Rank (Mantel-Cox) test (*p* = 0.23), and no significant difference was observed between the three groups.

### *Pv*SPECT1 showed sterile protection in 10% of mice after sporozoite challenge

To evaluate the *in vivo* protective efficacy of *Pv*SSP3 and *Pv*SPECT1, BALB/c (n = 10) mice were immunized with adjuvanted recombinant antigen and challenged with 500 *Pb-Pv*SSP3 or *Pb-Pv*SPECT1 sporozoites, respectively, and protection was assessed by monitoring blood-stage parasitemia. The antibody titers against r*Pv*SSP3 and r*Pv*SPECT1 were evaluated by endpoint ELISA. All immunized mice induced high-titer IgG antibodies against each antigen, with considerable variation in antibody responses between individual mice and endpoint titers ranging between 24,300–72,900 for r*Pv*SSP3 (S3A Fig) and 72,900–218,700 for r*Pv*SPECT1 (S3B Fig). No reactivity was observed with sera from mice immunized with TitreMax Gold adjuvant alone and pre-immune sera. In the r*Pv*SSP3 immunized group, 7 of 10 mice became patent on day 6, and 3 on day 7, compared with the adjuvant-only group, in which 9 of 10 mice became patent on day 6 and 1 on day 7, and 4 of 5 naïve mice on day 6 and 1 on day 7. The median pre-patency was 6 days across all three groups, and a Mantel-Cox (log-rank) test confirmed that there was no significant difference among the three groups of animals (*p* = 0.90) (Fig 6C). In the r*Pv*SPECT1 immunized group, 7 of 10 mice became patent on day 6, 2 on day 7, and 1 mouse remained free of parasitemia until day 14, indicating sterile protection. Patency was observed on day 6 in 9 of 10 mice in the adjuvant-only group and in all 5 naïve mice, with the remaining adjuvant-only mouse becoming patent on day 7. The median pre-patency was 6 days across all three groups, and a Mantel-Cox (log-rank) test confirmed that there was no significant difference among the three groups of animals (*p* = 0.23) (Fig 6D). Overall, both antigens had a limited impact on pre-patency following challenge, with r*Pv*SPECT1 inducing 10% sterile protection, whereas r*Pv*SSP3 provided none.

## Discussion

In this study, we generated transgenic rodent malaria parasites as a laboratory experimental model to study the *P. vivax* pre-erythrocytic antigens SSP3 and SPECT1 and as a resource to the malaria research community to evaluate their vaccine potential. Both antigens have desirable attributes as *P. vivax* PE vaccine candidates, based on prior functional studies linking them to sporozoite gliding motility, traversal activity, and EEF maturation, as well as preliminary immunological assessments [28,30,32–35]. Our previous study identified limited variation in predicted B- and T-cell epitopes of *Pv*SSP3 and *Pv*SPECT1 across geographically diverse *P. vivax* isolates [7], supporting their further evaluation as broad potential vaccine candidates. However, larger population-level studies will be needed to confirm their geographic coverage and assess the potential impact of naturally occurring sequence variation. Using a two-step GIMO-based transfection technology, we created two independent drug selectable marker-free *P. berghei* parasite lines that express either *Pv*SSP3 or *Pv*SPECT1. As observed in previous studies, not all human malaria parasite proteins, such as *P. falciparum* (Pf) P36, P52, B9, and SPECT2, introduced at their corresponding *P. berghei* gene locus, functionally complement their *P. berghei* orthologs to support parasite growth. Parasites expressing these Pf PE antigens showed normal growth in the blood and mosquito stages, but reduced liver stage infectivity and failed to initiate blood stage breakthrough [47,48]. So, we first assessed parasite fitness in both mice and mosquitoes across the life cycle. Our systematic evaluation showed that *Pv*SSP3 and *Pv*SPECT1 complemented the functions of their *P. berghei* orthologs in maintaining biological fitness during asexual blood-stage propagation in mice and their capacity to transmit to mosquito vectors. Quantitative analysis of midgut oocysts and salivary gland sporozoites confirmed that the *P. vivax* orthologs successfully supported development in the mosquito vector. The immunofluorescence data suggested that *Pv*SSP3 and *Pv*SPECT1 are expressed on sporozoites. The *in vitro* liver-stage progression analysis, *in vivo* liver burden, and pre-patency data suggested that the transgenic parasites exhibit normal growth during liver stages and achieve breakthrough blood stages on time, comparable to *Pb*WT parasites. Collectively, these findings demonstrate that *Pv*SSP3 and *Pv*SPECT1 complement the functions of *Pb*SSP3 and *Pb*SPECT1, respectively, in *P. berghei*.

Since our prior studies demonstrated that natural infections can elicit inhibitory antibodies to these antigens and antisera against these antigens can inhibit the hepatocyte invasion of *P. vivax* sporozoites [7,23], as a proof-of-concept, the transgenic parasite lines were evaluated for their vaccine potential using anti-SSP3 and anti-SPECT1 antibodies to inhibit sporozoite infection of hepatocytes *in vitro.* Antisera raised against recombinant *Pv*SSP3 and *Pv*SPECT1 modestly inhibited hepatocyte invasion by transgenic sporozoites *in vitro*, consistent with earlier observations [7,23]. The *Pv*SSP3 antiserum displayed a higher mean inhibition (16% to 43%) compared with the *Pv*SPECT1 antiserum (8% to 27%), suggesting differences in the relative contribution of each protein to the *in vitro* invasion process. Sporozoite gliding motility and traversal are important steps in establishing a successful liver stage infection [8]. When sporozoites are exposed to activating host factors, they are primed for productive invasion, and traversal through host cells has been shown to enhance infectivity [49,50]. SSP3 plays a key role in sporozoite gliding motility and EEF maturation [34,35], while SPECT1 is essential for crossing the sinusoidal layer and migration through the Kupffer cells to enter the liver [28]. In the *in vitro* system, the sporozoites have direct access to the hepatocytes. Incubation with serum or other host environmental factors activates sporozoites and primes them for hepatocyte infection, shifting from a traversal mode to productive invasion [49,51]. Incubation of sporozoites with mouse serum may activate a large portion of them, reducing the ability of antibodies in the serum to completely block hepatocyte invasion.

Despite measurable *in vitro* inhibition, r*Pv*SSP3 immunization did not confer detectable *in vivo* protection, whereas r*Pv*SPECT1 induced sterile protection in 1 of 10 mice with no significant difference in pre-patency. Analysis of pre-challenge sera showed that both immunizations elicited high antigen-specific antibody titers, although titers varied among individual mice, similar to what has been reported for naturally acquired antibodies in the serum of individuals from a malaria endemic region [23]. The discrepancy between *in vitro* activity and *in vivo* protection highlights that partial inhibition of hepatocyte invasion does not necessarily translate into sterilizing immunity after sporozoite challenge [52]. *Pv*SSP3 is composed of 448 amino acids, and the recombinant construct used to generate the sera corresponded to amino acids 19–203, which contains the P48/45 domain [7,23]. The lack of protection in mice immunized with r*Pv*SSP3 may be due to epitope specificity, in which few antibodies elicited by the selected region recognize the protective epitopes on the native protein in its physiological context, especially if important structural epitopes are lost or differently displayed in the shortened antigen [53]. Using the full-length recombinant *Pv*SSP3, eliminating the signal peptide and transmembrane region, to immunize mice may improve protective efficacy. Another reason for the limited protection by *Pv*SSP3 and *Pv*SPECT1 could be that the sporozoites were injected intravenously into mice at the time of challenge, thereby bypassing the skin phase of the parasite. Sporozoites need to cross several biological barriers in the vertebrate host to establish a successful liver stage infection [54,55]. Once released into the dermis by an infected mosquito, the *Plasmodium* sporozoite uses its gliding machinery to actively migrate through skin cells, rapidly reaching a nearby blood vessel and finally, the liver [8,56,57]. Sporozoite challenge by intravenous injection may not adequately reflect the immunity against antigens involved in gliding motility and traversal. This is because the intravenous route eliminates the skin phase of the infection, where antibodies can restrain sporozoite movement and thereby restrict vascular access [58,59]. In a recent study of Kenyan adults, R21/Matrix-M protected against *P. falciparum* sporozoite challenge via the intradermal route but not against direct venous inoculation, indicating that antibody-mediated protection is sensitive to the challenge route [60]. So, *Pv*SSP3 and *Pv*SPECT1 antigens may provide better protection when challenged either by mosquito bite or intradermal injection. It is also possible that stronger protection requires more potent vaccine delivery platforms, clinically relevant adjuvants, or multivalent antigen combinations that target complementary steps in sporozoite migration and hepatocyte entry [6,7]. Another possibility for the absence of detectable protection could be due to TitreMax Gold, an experimental adjuvant commonly used in animal studies, but this formulation may not fully reflect a clinically translatable vaccine platform. Future studies are warranted to assess clinically relevant adjuvants to improve their vaccine potential.

Overall, these findings establish the *Pb-Pv*SSP3 and *Pb-Pv*SPECT1 lines as robust tools for systematically evaluating SSP3 and SPECT1 as *P. vivax* pre-erythrocytic vaccine candidates. Rodent models provide a tractable platform for down-selecting antigens, comparing delivery systems and alternative routes such as direct mosquito bite, intradermal, or subcutaneous injections, identifying compatible adjuvants, and identifying antigens that induce broad cross-strain protection. They enable the identification of immune correlates of protection by allowing mechanistic analysis of antibody and T-cell responses before proceeding to primate and human clinical trials. The integration of surrogate rodent models with advanced technologies offers a clear pathway to develop the next generation of vaccines needed to eliminate *P. vivax*.

In conclusion, our study establishes that *Pv*SSP3 and *Pv*SPECT1 functionally complement their rodent orthologs and can be successfully expressed in a transgenic *P. berghei* system. These transgenic parasites are marker-free and are amenable to further genetic manipulations. These transgenic parasites provide a platform for replacing additional *P. berghei* proteins with their *P. vivax* orthologs and to evaluate multivalent pre-erythrocytic vaccine designs. Future studies would explore different antigen-delivery platforms and clinically relevant adjuvant formulations to enhance immunogenicity and protective efficacy of these antigens, thereby supporting the development of strategies to eliminate *P. vivax* malaria.

## Supporting information

S1 Fig Immunofluorescence staining of *Pb-Pv*SSP3 and *Pb-Pv*SPECT1 salivary gland (SG) sporozoites using pre-immune mouse serum. mCherry expressing *Pb-Pv*SSP3 (top panel) and GFP expressing *Pb-Pv*SPECT1 (bottom panel) SG sporozoites were stained with pre-immune mouse serum and revealed with Alexa Fluor 488-conjugated anti-mouse secondary antibody (top panel) or Alexa Fluor 594 conjugated anti-mouse secondary antibody (bottom panel). Nuclei were stained with Hoechst 33342. Scale bar - 5 μm.
(TIFF)

S2 Fig Parasite liver loads of *Pb-Pv*SSP3 and *Pb-Pv*SPECT1 lines in mice. Ten thousand luciferase expressing sporozoites of *Pb*WT, *Pb-Pv*SSP3, or *Pb-Pv*SPECT1 were injected into Swiss Webster ND4 mice (n = 5), and the parasite liver loads were measured at 44 h post infection. The luciferase activities of the *Pb*WT, *Pb-Pv*SSP3, and *Pb-Pv*SPECT1 parasites were measured using the IVIS Lumina II Imaging System.
(TIFF)

S3 Fig Immunogenicity of r*Pv*SSP3 and r*Pv*SPECT1 in BALB/c mice. BALB/c mice (n = 10) were immunized with r*Pv*SSP3 or r*Pv*SPECT1 formulated with TiterMax Gold adjuvant. Before challenge, serum from each mouse was evaluated by endpoint-titer ELISA for antibodies recognizing r*Pv*SSP3 (**A**) or r*Pv*SPECT1 (**B**). Each curve represents an individual mouse. Sera from mice that received TiterMax Gold alone and pre-immune sera (PI) served as controls. Error bars indicate standard deviation.
(TIFF)

S1 Table List of primers. (PDF)
