# Supplementary material for: Transgenic Plasmodium berghei expressing P. vivax pre-erythrocytic antigens SSP3 and SPECT1 to elucidate their efficacy towards vaccine development
Source: PLoS Negl Trop Dis. 2026 Oct 22;20(10):e0014810. doi: 10.1371/journal.pntd.0014810 (PMC13739220; doi:10.1371/journal.pntd.0014810)
Supplement: Supplementary file 1 [file pntd.0014810.s004.pdf]

S1 Table: List of primers

Primers used to generate targeting constructs

DNA Construct	Primer code	Sequence	Enzymes	Product (bp)	Primer description
SK013/ pL2381	JA-4374	CACAAGCTTGGGATTCTTAAAATGTTCACTATTCAC	HindIII	950 bp	*Pbssp3* 5'HR forward
	JA-4375	GACCGCGGATCCATTTTAATAGCGTGATGTAAAAGAAAGGG	SacII, BamHI		*Pbssp3* 5'HR reverse
	JA-4376	GCAGGTACCATAAAACATCAAAATAATGGATAAAGAAG	KpnI	1053 bp	*Pbssp3* 3'HR forward
	JA-4377	CGAGCGGCCGCGAACCATTGTTACTCTCCCCATGATAT	NotI		*Pbssp3* 3'HR reverse
pL2157	AS-1256	GCACCTACGTATTTGGTTGATGGTGCTTATATATCAAACTG	ApaI	1425 bp	*Pbspect1* 5'HR forward
	AS-1257	GCTCCGCGGTGTGTTAAACGATATGTGATAATTAATGAGG	SacII		*Pbspect1* 5'HR reverse
	AS-1258	TGTCCCGGGACATCTATATATCAGTTCGAAATTTGAAC	xmaI	1311 bp	*Pbspect1* 3'HR forward
	AS-1259	GTAGCGGCCGCTATATATTATGTATGGATTATGGTGCTCATAGATTAT	NotI		*Pbspect1* 3'HR reverse
SK017/ pL2383	JA-4612	CACACCGCGGATGGAACTCTTCACTTCCCTTC	SacII	746 bp	*Pvspect1 orf* forward
	JA-4613	TCCCCTCGAGCTATATTATTTTCCCCAAATCGGCGG	XhoI		*Pvspect1 orf* reverse
SK025/ pL2390	JA-4439	*CTTTCTTTTACATCACGCTATTAAAAT*ATGTCGAACCTCGCCCGCTAC	-	1402 bp	*Pvssp3 orf* forward
	JA-4440	*ATCCATTATTTTGATGTT*TTATGGTACCTCACAACACGTCGGACGAC	-		*Pvssp3 orf* reverse

Primers used for integration PCRs

Line Number	Primer code	Sequence	Product size (bp)	Primer description	Product details
*Pb*SSP3-GIMO 3374cl1	JA-5050	CTACTTATACAAAAAAAGCAAATATATCATGAA TACTG	1182	*Pbssp3* integration forward	*5'* integration
	JA-4893	GGGTGAGCATTTAAAGCACAATATCTAGG		*Pbef1a* promoter reverse	
	JA-4894	GTCTCTTCAATGATTCATAAATAGTTGGACTTG	1328	*Pbdhfr 3'utr* forward	*3'* integration
	JA-4662	CCCCTTTTGTCATAGATGAATTCGGAACCC		*Pbssp3* integration reverse	
	JA-4794	GGTATTCCACGCTGTGTTGTCGGTGAG	947	SM forward	*selectable marker*
	JA-4795	GTTCATAATTTTAGCTATTTACATGCATGTGC		SM reverse	
	JA4659	GCATGAAATAGGAAATGGAGATAGTATAGG	945	*Pbssp3 orf* forward	Absence of *Pb ssp3 orf*
	JA-4660	CCTTCCATTAACCGATATCCATTTCC		*Pbssp3 orf* reverse	

Line Number	Primer code	Sequence	Product size (bp)	Primer description	Product details
*Pb-Pv*SSP3 3395cl1	JA-4665	ATGTCGAACCTCGCCCGCTACTT	814	*Pvssp3 m2 orf* forward	*Pv ssp3 orf*
	JA-4666	TGGATTTTATACTTGGGGGTCTGGCTTC		*Pvssp3 m2 orf* reverse	
	JA-5050	CTACTTATACAAAAAAAGCAAATATATCATGAATACTG	1186	*Pbssp3* integration forward	*5'* integration
	JA-4462	CAATTTCGTGGTAGCACGTGACATC		*Pvssp3* integration reverse	
	JA-4461	GTCATCTTCGCCTGGACCTCCATTG	1345	*Pvssp3* integration forward	*3'* integration
	JA-4662	CCCCTTTTGTCATAGATGAATTCGGAACCC		*Pbssp3* integration reverse	
	JA-4794	GGTATTCCACGCTGTGTTGTCGGTGAG	947	SM forward	Absence of *selectable marker*
	JA-4795	GTTCATAATTTTAGCTATTTACATGCATGTGC		SM reverse	
	JA-4659	GCATGAAATAGGAAATGGAGATAGTATAGG	945	*Pbssp3 orf* forward	Absence of *Pb ssp3 orf*
	JA-4660	CCTTCCATTAACCGATATCCATTTCC		*Pbssp3 orf* reverse	
*Pb*SPECT1-GIMO 2672cl2	JA-4555	CACACATGCAAGTATGTATAAAAATACCGTG	1646	*Pbspect1* integration forward	*5'* integration
	JA-4893	GGGTGAGCATTTAAAGCACAATATCTAGG		*Pbef1a* promoter reverse	
	JA-4894	GTCTCTTCAATGATTCATAAATAGTTGGACTTG	1634	*Pbdhfr 3'utr* forward	*3'* integration
	JA-4556	CAAAATAGCACAGAATTCCCTTTAACATGCC		*Pbspect1* integration reverse	
	JA-4794	GGTATTCCACGCTGTGTTGTCGGTGAG	947	SM forward	*selectable marker*
	JA-4795	GTTCATAATTTTAGCTATTTACATGCATGTGC		SM reverse	
	JA-4557	GATGTCCACCATTGTGGATCCTC	902	*Pbspect1 orf* forward	Absence of *Pb spect1 orf*
	JA-4558	CGTCGTTGTTCTTTATTGATTCTAAAACCCCC		*Pbspect1 orf* reverse	
*Pb-Pv*SPECT1 3378cl1	JA-4645	ATTCGAAGAAAGAGACGTACTCTCAC	655	*Pvspect1 orf* forward	*Pv spect1 orf*
	JA-4646	CAAATCGGCGGAAACTTCTCGTATTAAAGC		*Pvspect1 orf* reverse	
	JA-4555	CACACATGCAAGTATGTATAAAAATACCGTG	1602	*Pbspect1* integration forward	*5'* integration
	JA-4643	CTTATCCACGTGTGAGAGTACGTCTC		*Pvspect1* integration reverse	
	JA4644	CATATCTGATGAGCTAAAAAGCGAGCTG	1661	*Pvspect1* integration forward	*3'* integration
	JA-4556	CAAAATAGCACAGAATTCCCTTTAACATGCC		*Pbspect1* integration reverse	
	JA-4794	GGTATTCCACGCTGTGTTGTCGGTGAG	947	SM forward	Absence of *selectable marker*
	JA-4795	GTTCATAATTTTAGCTATTTACATGCATGTGC		SM reverse	
	JA-4557	GATGTCCACCATTGTGGATCCTC	902	*Pbspect1 orf* forward	Absence of *Pb spect1 orf*
	JA-4558	CGTCGTTGTTCTTTATTGATTCTAAAACCCCC		*Pbspect1 orf* reverse	

orf - open reading frame; HR - homology region and SM - selectable marker
